# Clinical Applications of Orthodontic Mini-Implants in Orthodontic and Esthetic Practice: A Case Series Exploring Biomechanical Versatility

**DOI:** 10.3390/dj14030132

**Published:** 2026-02-25

**Authors:** Teodora Consuela Bungău, Ada Radu, Gabriela Ciavoi

**Affiliations:** 1 Doctoral School of Biomedical Sciences, University of Oradea, 410087 Oradea, Romania; consuela.bungau@uoradea.ro; 2 Department of Dentistry, Faculty of Medicine and Pharmacy, University of Oradea, 410073 Oradea, Romania; 3 Department of Pharmacy, Faculty of Medicine and Pharmacy, University of Oradea, 410028 Oradea, Romania

**Keywords:** mini-implants, orthodontic, case series, anchorage

## Abstract

**Background/Objectives:** Orthodontic mini-implants have become indispensable in modern orthodontics due to their ability to provide absolute anchorage, independent of patient compliance. Our research aims to illustrate the versatility of mini-implants in addressing diverse biomechanical challenges across different planes of tooth movement (sagittal, transverse, and vertical) based on a retrospective clinical analysis. **Methods**: A retrospective analysis of orthodontic treatments performed with mini-implants (Dual Top and JS systems) was conducted, focusing on predefined biomechanical objectives and outcomes. The analysis encompassed distinct biomechanical applications, including incisor retraction and space closure using sequential direct and indirect anchorage; transverse and vertical correction of adult open bite through mini-implant–assisted rapid palatal expansion (MARPE) and molar intrusion; deep bite correction via simultaneous upper and lower incisor intrusion; and unilateral molar distalization using palatal skeletal anchorage. **Results**: Mini-implants provided stable, reproducible anchorage in all cases, enabling complex three-dimensional tooth movements with minimal side effects. Sequential reuse of the same mini-implants for both indirect and direct anchorage reduced treatment invasiveness and enhanced anchorage efficiency. Combined skeletal expansion and posterior intrusion allowed improved transverse and vertical control in adult open-bite presentations. Pure incisor intrusion was achieved without molar extrusion or incisor proclination, while unilateral molar distalization was effectively managed using palatal skeletal anchorage. Across all cases, mini-implants enhanced treatment efficiency, reduced the need for auxiliary appliances, and ensured predictable outcomes. **Conclusions**: Orthodontic mini-implants represent a highly versatile and minimally invasive anchorage system adaptable to a broad range of biomechanical situations. Their ability to provide stable, reusable, and site-specific anchorage supports efficient correction of complex malocclusions and reinforces their pivotal role in contemporary orthodontic practice.

## 1. Introduction

In recent decades, orthodontic mini-implants have revolutionized the concept of anchorage control, offering orthodontists new possibilities to perform personalized, efficient, and biomechanically sound treatments [1]. Their small dimensions, ease of insertion, minimally invasive nature, and affordable costs have turned them into an indispensable tool in contemporary orthodontics [2]. Unlike traditional anchorage methods, which often rely on patient compliance or carry the risk of unwanted side effects, mini-implants provide maximum anchorage, allowing for precise and predictable tooth movements that were previously difficult to achieve [3].

The versatility of mini-implants is not limited to their mechanical properties but is also reflected in their wide range of clinical applications. They can be used for both direct and indirect anchorage, facilitating movements such as intrusion, extrusion, distalization, or mesialization, and significantly contributing to the correction of vertical and sagittal discrepancies [4]. Their adaptability allows them to be integrated into various treatments, from simple space closures to complex interdisciplinary cases involving surgical or prosthetic components [5].

Furthermore, mini-implants enhance treatment efficiency by reducing overall duration, eliminating the need for extraoral devices, and, in many cases, avoiding dental extractions or compensatory mechanics. This brings benefits both to the clinician, through increased control and predictability, and to the patient, by improving overall management [6].

The use of mini-implants has transformed orthodontic therapy by enabling clinicians to achieve superior biomechanical control with minimal reliance on patient cooperation. This advancement not only streamlines treatment protocols but also enhances consistency in clinical outcomes. From a patient-centered standpoint, assessments of mini-implant applications predominantly address experiential factors, such as comfort during therapy, the intensity of postoperative discomfort, and overall satisfaction throughout the orthodontic process [7].

The present research aims to analyze and synthesize the clinical versatility and biomechanical advantages of orthodontic mini-implants in contemporary practice, based on retrospective clinical applications addressing predefined biomechanical objectives. The reported treatments illustrate how mini-implants can be strategically integrated to optimize treatment efficiency, achieve controlled and complex tooth movements, and preserve anchorage integrity across sagittal, transverse, and vertical planes of space. By demonstrating their capacity to replace or enhance conventional anchorage methods in different biomechanical contexts, mini-implants are shown to expand the therapeutic scope of orthodontics, reducing treatment time, minimizing invasiveness, and improving both predictability and patient comfort. Collectively, these clinical applications support the role of mini-implants as a cornerstone of modern orthodontic biomechanics and as a key enabler of individualized, high-precision treatment outcomes.

## 2. Materials and Methods

### 2.1. Patient Selection and Data Collection

To illustrate the applicability and versatility of orthodontic mini-implants in clinical practice, several cases treated in the first author’s private practice were selected, in which skeletal anchorage was used as a key element in achieving the therapeutic objectives. The inclusion criteria targeted patients undergoing active orthodontic treatment, for whom the use of mini-implants was indicated to obtain stable anchorage necessary for specific tooth movements. In total, four patients (four clinical applications) were included. Given the retrospective, practice-based nature of this study, the unit of analysis was defined as the biomechanical application and its outcome domain (sagittal, transverse, vertical correction, and anchorage strategy), rather than a stand-alone case narrative. Accordingly, patient descriptors and miniscrew-related parameters were collected to support traceability and reproducibility of the applied mechanics.

The case selection was performed retrospectively, from a clinical database (Dental Art, Oradea, Romania), focusing on the diversity of clinical situations and the biomechanical relevance of the skeletal anchorage used. For each included case, the treatment plan, therapeutic objectives, mini-implant positioning, type of forces applied, clinical evolution, and outcome were analyzed.

Four cases were selected to include patients of different ages and to represent a range of clinical scenarios, in which mini-implants were used to facilitate tooth movements in different spatial planes (i.e., sagittal, vertical, and transverse). Skeletal diagnosis was reported according to available clinical and cephalometric data. Because of the retrospective and illustrative nature of the cases presented, baseline variables were not collected in a standardized manner across all patients. Table 1 summarizes the baseline characteristics and miniscrew-related information of the presented cases.

### 2.2. Mini-Implant Systems, Insertion Protocol, and Loading Approach

**Figure 1:**
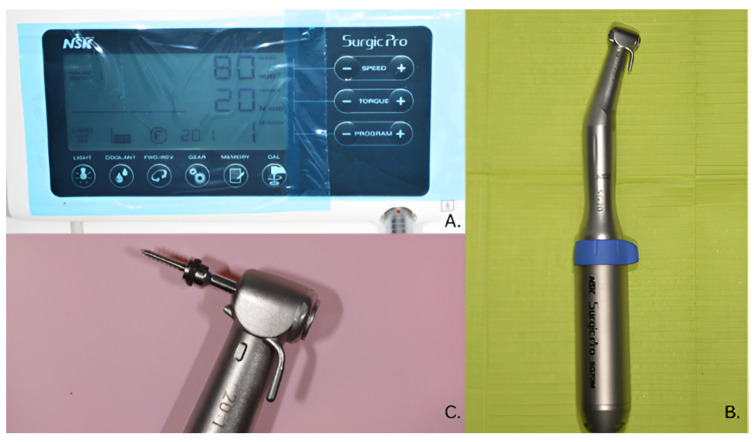
(**A**) Surgic Pro physiodispenser (NSK), (**B**) NSK low-speed rotary handpiece, (**C**) Dual Top insertion driver and mini-implant.

In the clinical applications analyzed in this study, self-drilling mini-implants from the Dual Top and JS systems (Jeil Medical, Seoul, Republic of Korea) were used due to their mechanical reliability and clinical adaptability across multiple anchorage protocols [8,9]. The insertion was per-formed under local anesthesia using rotary instrumentation—specifically, the Surgic Pro NSK physiodispenser (NAKANISHI INC, Kanuma, Japan) (Figure 1)—a method that al-lows improved control of torque and insertion.

Orthodontic loading of the mini-implants was carried out immediately after verifying primary stability post-insertion, an essential criterion for maintaining anchorage control. Details regarding insertion torque, insertion speed, and duration of loading for each case are presented in Table 2. The insertion site was individualized according to the specific clinical situation, the type and direction of the desired tooth movements, and was determined based on CBCT imaging and panoramic radiography evaluations.

In all treatments included in this analysis, the selection of mini-implant dimensions and insertion sites was performed in accordance with current recommendations from the scientific literature and will be detailed specifically within each presented case. Throughout treatment, all mini-implants demonstrated clinical stability throughout the active treatment phase, with no mobility observed during the loading period. Minor soft-tissue complications were noted in cases involving vestibular placement, primarily associated with suboptimal oral hygiene; however, these events did not compromise mini-implant stability or treatment progression.

It should be noted that for each patient, the analysis focuses strictly on the use of the orthodontic mini-implant and the intended tooth movement, without including a detailed description of the general treatment plan or the complete evolution of the case, as these aspects are beyond the scope of the present article.

### 2.3. Ethical Considerations

The patients whose cases are presented had previously signed an informed consent form regarding orthodontic treatment, as well as an explicit agreement concerning the processing of personal data (GDPR) and allowing the use of their clinical images for scientific, educational, and research purposes.

All procedures related to data collection and use were conducted in accordance with current ethical standards. The case study was approved by the Research Ethics Subcommittee of the University of Oradea, under Approval No. 4459/28 March 2025 and in respect of the Helsinki Agreement for studies implying human [10].

To protect patient identity and ensure confidentiality, only intraoral images (belonging to the personal archive of the first author) were used in this work, without including facial images or other personal identifying data.

The following section synthesizes these clinical applications into a coherent analytical framework, highlighting outcome-oriented biomechanical strategies rather than isolated case descriptions.

## 3. Results

In this retrospective analysis (n = 4 patients), outcomes are reported by predefined clinical/biomechanical domains reflecting the primary objectives achieved with skeletal anchorage. Case identifiers (Case 1–4) are retained exclusively for clinical traceability and linkage to the corresponding figures; the Results are therefore structured by outcome domain rather than as stand-alone case narratives.

### 3.1. Sagittal Correction Outcomes (Anterior Retraction and Distalization)

This subsection reports sagittal tooth movement achieved through mini-implant–supported biomechanics, including anterior segment retraction and unilateral molar distalization, as illustrated in two clinical applications (corresponding to Cases 1 and 2).

**Figure 2:**
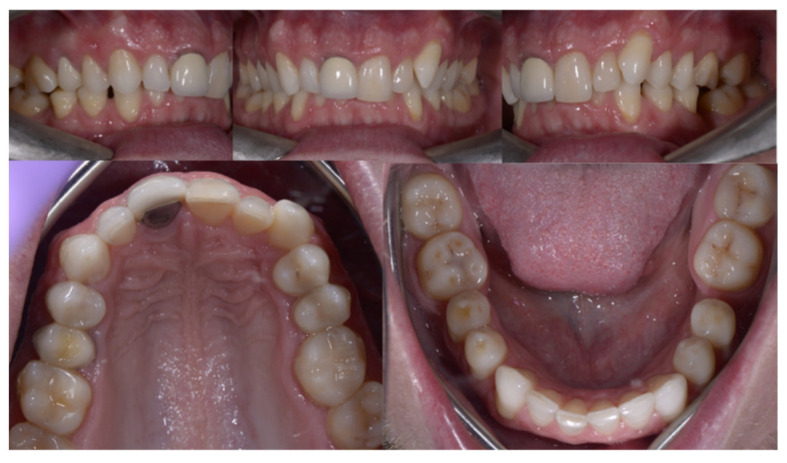
Initial intraoral photographs.

A 34-year-old patient (Case 1) presented to the clinic with the specific request to replace the dental crown on tooth 11, being dissatisfied both esthetically and functionally. Clinical examination and radiographic analysis revealed a Class II, subdivision II malocclusion, deep bite, and dento-maxillary disharmony with crowding. Additionally, posterior tooth loss in the mandibular arch was observed in the areas of teeth 36 and 46, accompanied by a reduced interproximal space (Figure 2).

In this context, a comprehensive orthodontic treatment was proposed to the patient, aiming to correct occlusal relationships, improve masticatory function, and achieve stable occlusal contacts that would later allow for the esthetic restoration of the anterior region.

Treatment objectives included:

- Correction of canine distalization relationships to achieve proper canine guidance;
- Correction of upper incisor retrusion;
- Correction of deep bite;
- Closure of existing spaces in the mandibular arch;
- Establishment of optimal morpho-functional and esthetic balance;
- Prosthetic rehabilitation of the upper anterior region;
- Maintenance of treatment results through retention.

The treatment plan involved:

- Application of a self-ligating orthodontic appliance;
- Extraction of teeth 15 and 24 to create the necessary space for incisor retraction;
- Use of orthodontic mini-implants to obtain optimal anchorage, implemented in two distinct biomechanical phases.

**Figure 3:**
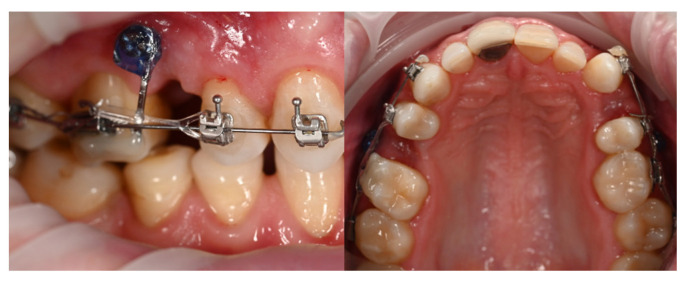
Application of the bilateral segmented appliance. Indirect anchorage to the mini-implant.

Treatment began with the bonding of a bilateral segmented appliance, extending from the canine to the second molar. Two vestibular G2 mini-implants measuring 1.6 × 10 mm were inserted medially to teeth 16 and 26, positioned at the junction between the attached and movable mucosa (Figure 3).

The mini-implants were used to indirectly anchor the upper first molars through a customized 0.019 × 0.025-inch stainless steel segmented arch, bonded directly to the teeth with flowable composite. To protect the buccal mucosa, the head of each mini-implant was covered with liquid dam material.

An elastic chain was applied from 14 to 16 and from 23 to 26 to distalize the premolar and canine. This stage was completed once the canines reached their desired position, required for initiating anterior retraction.

**Figure 4:**
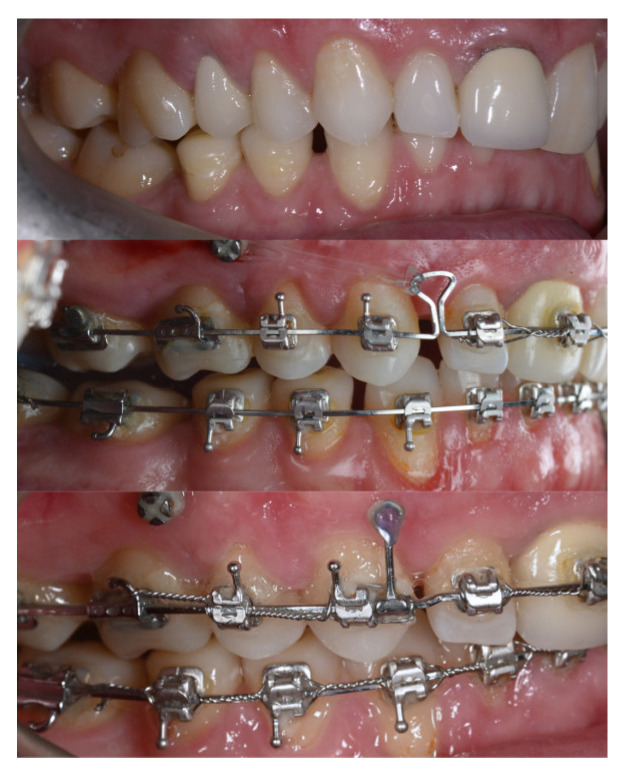
Retraction of the incisor segment for space closure using direct anchorage.

**Figure 5:**
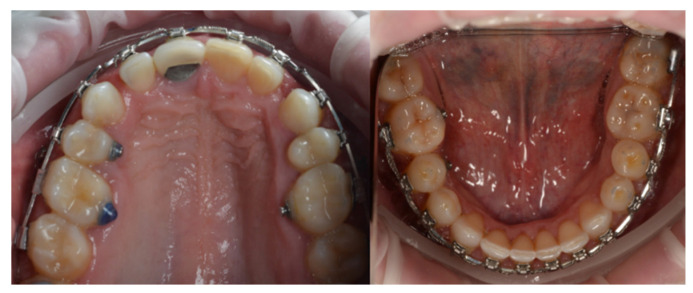
Intraoral images showing treatment progress.

After completing the distalization phase, the orthodontic appliance was bonded across the entire arch, initiating the alignment and leveling stage. Once the rectangular working archwires with closing loops were engaged, retraction of the upper incisor segment began by applying elastic chains from the loops of the archwire to the anteriorly placed mini-implants. In this way, the same mini-implants were used sequentially for both indirect and direct anchorage, demonstrating the clinical versatility of these devices (Figure 4 and Figure 5).

**Figure 6:**
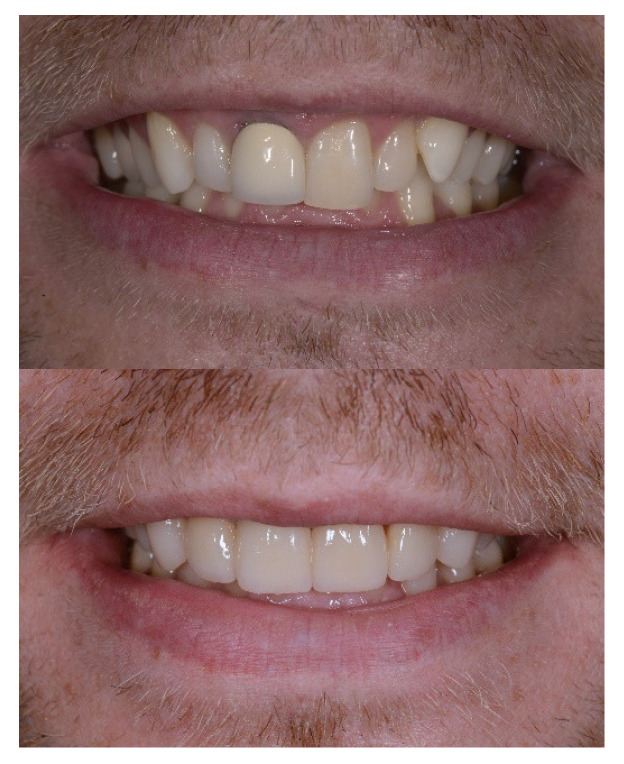
Before and after case completion with veneering of the upper incisors.

At the end of orthodontic treatment, the patient underwent veneering of the four upper incisors to achieve esthetic harmony of the smile and functional integration of the initially planned prosthetic restoration, as depicted in Figure 6.

**Figure 7:**
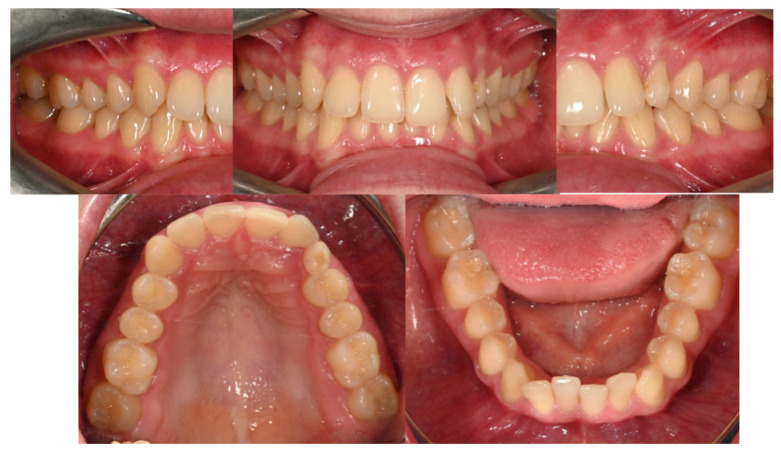
Intraoral photographs—initial situation.

Sagittal correction using skeletal anchorage was also applied in a 22-year-old patient (Case 2) presenting with unilateral space deficiency associated with an impacted canine. Clinical examination and radiographic analysis revealed persistence of tooth 63 and palatal impaction of tooth 23, associated with a slight generalized mesial displacement of the upper left hemiarch (Figure 7).

Treatment objectives were as follows:

- Alignment and leveling of both arches;
- Unilateral distalization of molar 26 to create the necessary space for the impacted canine;
- Surgical exposure of canine 23 and controlled traction into the arch;
- Finishing of the occlusion and retention of the orthodontic result.

The treatment plan included: a bimaxillary self-ligating fixed orthodontic appliance was selected, with a band cemented on tooth 26 that included an anterior palatal hook designed to facilitate the application of distalizing force. For skeletal anchorage, a palatal JS mini-implant measuring 2 × 10 mm was inserted mesially to tooth 26 in the interradicular area. The mini-implant was placed at a slight oblique angle to allow subsequent distal movement of the premolars without interference.

**Figure 8:**
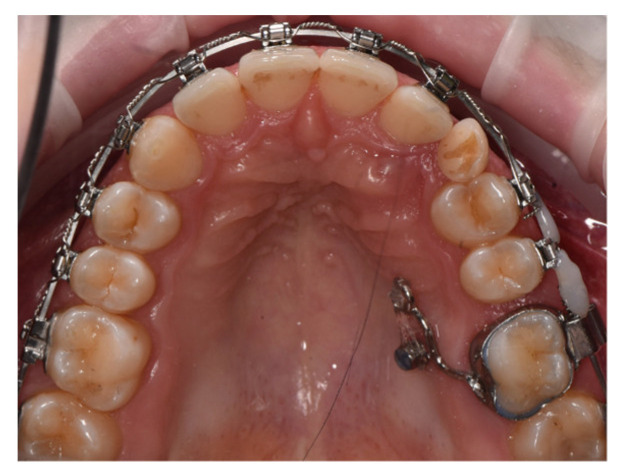
Distalization of the upper left first molar using direct anchorage—space gained after two activations.

**Figure 9:**
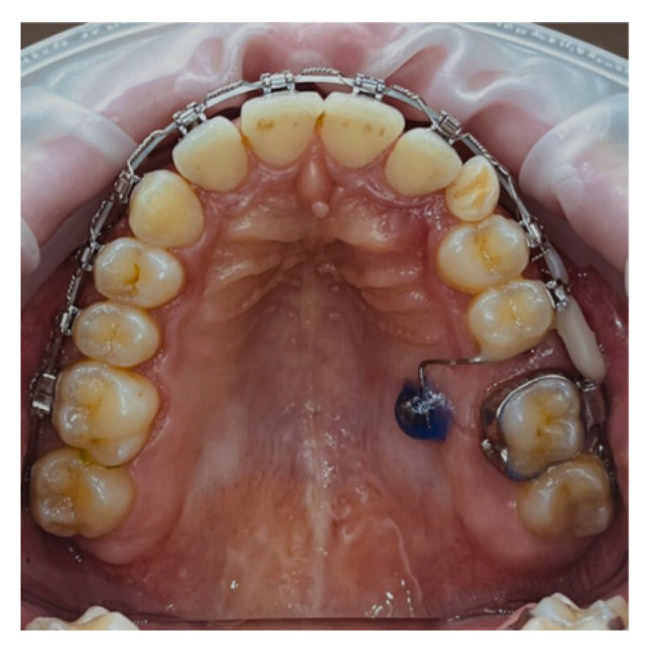
Use of the palatal mini-implant for indirect anchorage of tooth 25. Distalization continued with a NiTi open-coil spring applied buccally on the main archwire.

Initially, the distalizing force on the molar was applied using an elastic chain stretched between the anterior palatal hook and the mini-implant. Figure 8 shows the space gained after two activations. Unfortunately, after several activations, the hook fractured, requiring a change in biomechanical strategy. An indirect anchorage approach was then adopted by stabilizing the second premolar (25) with a 0.019 × 0.025-inch stainless steel segmented wire, rigidly connected to the mini-implant. This setup allowed distalization of molar 26 to continue using a NiTi open-coil spring, while preventing unwanted tipping effects on the premolar, which remained passive (Figure 9).

**Figure 10:**
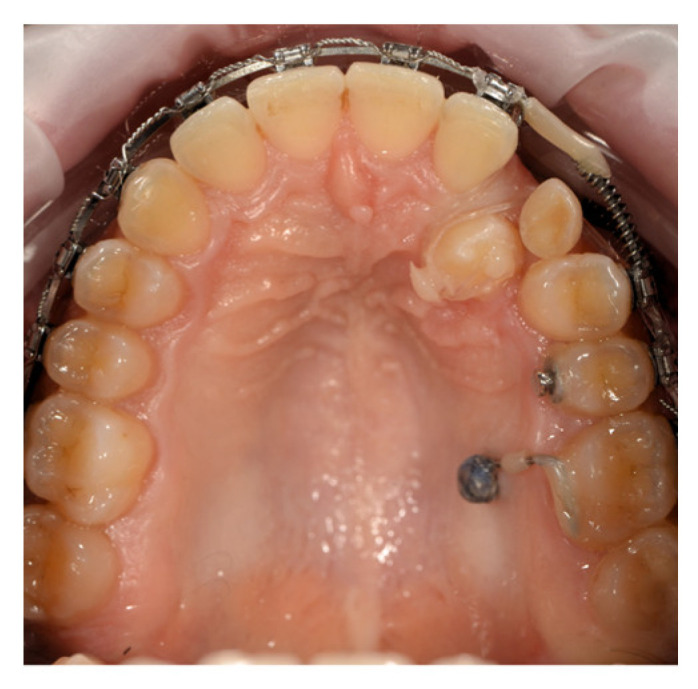
Stabilization of tooth 26 to the mini-implant with a segmented wire and continued distalization of the premolars to create space for tooth 23. Surgical exposure of tooth 23.

After achieving the desired distalization, the same mini-implant was used to maintain the newly obtained molar position, serving as indirect anchorage for the subsequent distalization of the premolars. Once adequate space was created for tooth 23, surgical exposure of the impacted canine was performed, followed by its progressive traction into the arch using controlled forces (Figure 10).

Across the analyzed clinical applications, orthodontic mini-implants demonstrated the capacity to be employed sequentially as both indirect and direct anchorage units within the same treatment protocol, depending on the evolving biomechanical requirements. This adaptability represents a key functional advantage of skeletal anchorage systems and was most clearly observed in the sagittal correction strategies described in this series.

#### Sequential Anchorage Strategy Within Sagittal Correction Protocols

In the sagittal correction protocol corresponding to Case 1, vestibular mini-implants were initially utilized to provide indirect anchorage, stabilizing the posterior dentoalveolar segment during premolar and canine distalization (Figure 3). Following completion of this phase, the same mini-implants were subsequently engaged as direct anchorage units to support anterior segment retraction (Figure 4 and Figure 5), without the need for reinsertion or modification of the skeletal anchorage site.

A comparable sequential anchorage strategy was applied in the unilateral distalization protocol corresponding to Case 2. In this application, a palatal mini-implant initially functioned as a direct anchorage unit for molar distalization (Figure 8). Subsequently, the anchorage configuration was modified, and the same mini-implant was incorporated as an indirect anchorage unit to stabilize the premolar segment and maintain the achieved sagittal correction (Figure 9).

These sequential anchorage strategies highlight the adaptability of mini-implants in responding to changing biomechanical requirements during treatment, while minimizing invasiveness and maintaining anchorage integrity.

### 3.2. Transverse and Vertical Correction Outcomes

This subsection describes combined transverse skeletal expansion and vertical posterior intrusion achieved with mini-implant–assisted biomechanics in an adult open-bite presentation (Case 3).

**Figure 11:**
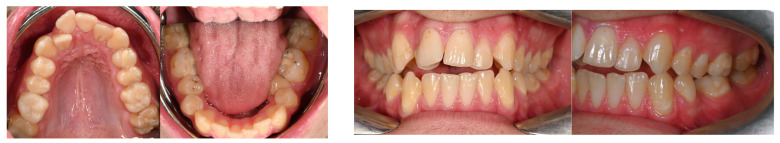
Intraoral photographs—initial situation.

A 27-year-old patient presented for orthodontic consultation, reporting both functional difficulties and esthetic concerns. Clinical examination and radiographic analysis established the following orthodontic diagnosis:

- Narrow maxilla;
- Anterior open bite;
- Severe deviation of the upper midline to the right;
- Ectopic canine (13) and malpositioned lateral incisor (12);
- Dento-maxillary discrepancy with severe crowding (Figure 11).

In addition, the patient exhibited atypical swallowing and oral breathing, for which speech therapy sessions and myofunctional breathing exercises were recommended.

Following interdisciplinary evaluation and considering the patient’s dento-skeletal and functional characteristics, the following treatment objectives were established:

- Mini-implant-assisted maxillary expansion;
- Intrusion of the posterior segments to close the anterior open bite;
- Correction of the midline deviation;
- Creation of space through expansion and strategic extractions;
- Alignment and leveling of both arches;
- Stabilization and retention of the orthodontic outcome.

The treatment plan was structured into several stages, including the following procedures:

- Extractions of teeth 35 and 24 to create the necessary space;
- Corticotomy and corticopuncture to facilitate expansion;
- Intrusion of the upper molars using mini-implants and segmented arches;
- Placement of a fixed orthodontic appliance.

**Figure 12:**
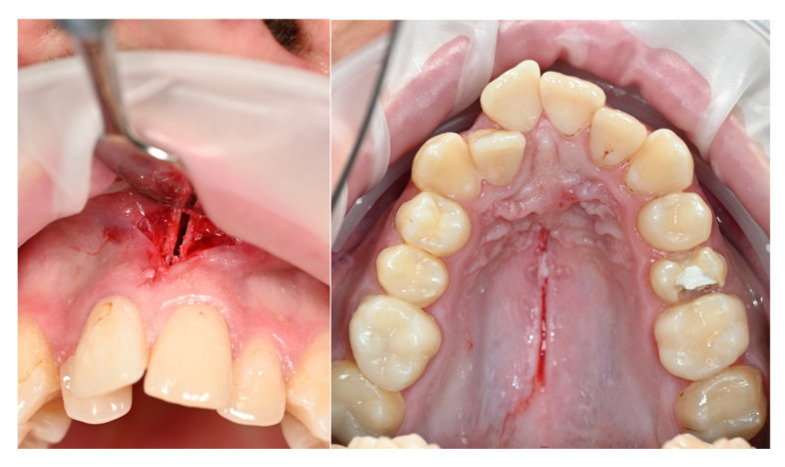
Incisions for corticotomy and corticopuncture.

**Figure 13:**
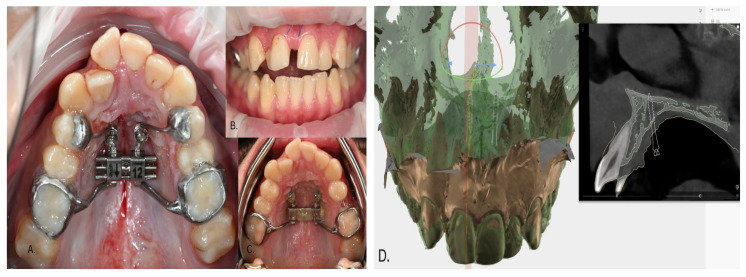
(**A**) Cementation of the expander and insertion of the mini-implants. (**B**) Appearance of the interincisal diastema one week after activation. (**C**) Locking of the expander and maintenance in the palate for retention of the result. (**D**) Digital planning of mini-implant insertion.

The initial phase included corticotomy and corticopuncture (Figure 12) along the mid-sagittal suture, followed by the placement of a mini-implant-supported expander (Figure 13A). Two JS orthodontic mini-implants, measuring 2 × 12 mm, were inserted in the palatal region, according to the digital planning (Figure 13D). After achieving the desired expansion, the expander was locked and maintained in position as a passive retention device (Figure 13C).

**Figure 14:**
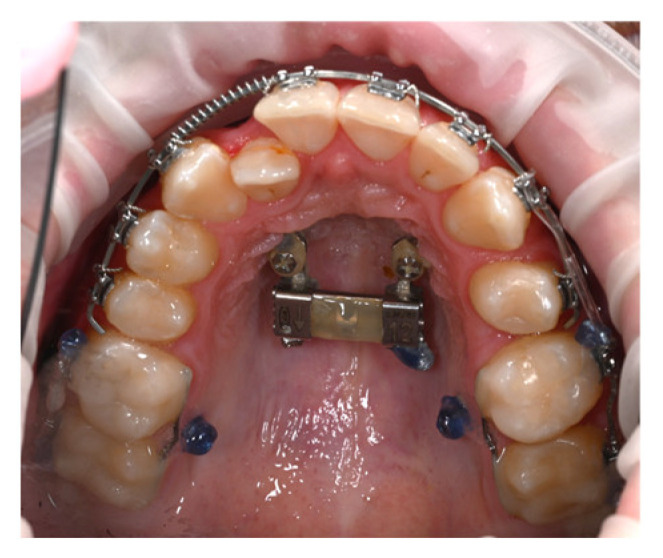
Intraoral photograph during treatment—upper fixed orthodontic appliance, segmented arches in the posterior areas, and mini-implants for intrusion.

Three months post-expansion retention, the arms of the expander and the corresponding molar bands were removed, and a fixed self-ligating appliance was bonded on the upper arch. The treatment was biomechanically divided into two distinct segments:

- The premolar-to-premolar segment, treated with a continuous orthodontic archwire for alignment and leveling.
- The posterior segments, treated separately using vestibular and palatal segmented stainless steel (SS) archwires of 0.019 × 0.025 inch, manually adapted to deliver intrusive forces (Figure 14).

For the intrusion of the upper molars, four orthodontic mini-implants were inserted in an interradicular position:

- Two buccal mini-implants (1.6 × 10 mm, JA) placed between teeth 16–17 and 26–27;
- Two palatal mini-implants (2 × 10 mm, JS) positioned between the same teeth.

This configuration allowed for efficient intrusion of the posterior segments through symmetrical application of intrusive forces, providing axial control on both the buccal and palatal aspects of the molars. As a result, unwanted buccal or lingual tipping of the crowns was prevented. The intrusive forces were applied using elastic chains extending from the mini-implant heads to the segmented archwires.

**Figure 15:**
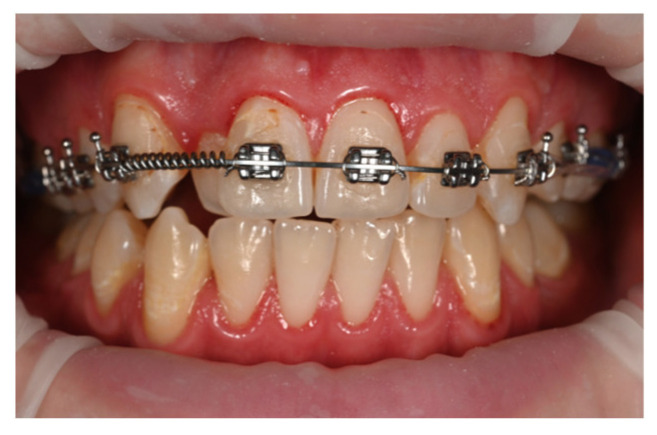
Intraoral photograph during treatment—progressive closure of the anterior open bite.

As illustrated in Figure 15, intrusion of the posterior segments was effective, with visible improvements observed even in the early stages of force application. The progressive closure of the anterior open bite confirmed the efficiency of the chosen biomechanical protocol and highlighted the importance of dual anchorage (buccal and palatal) in achieving stable and well-directed tooth movements.

**Figure 16:**
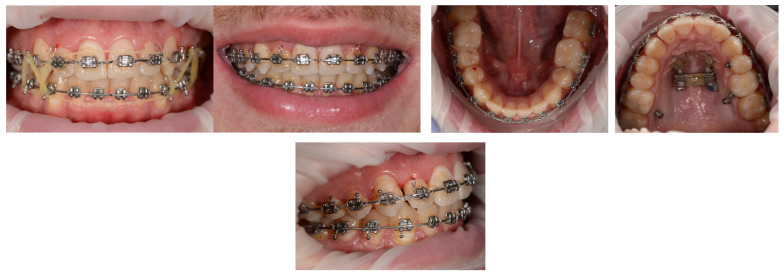
Clinical aspect during the finishing stage.

Orthodontic mini-implants played a key role in the success of the treatment, being used both for transverse modifications (through maxillary expansion) and for vertical control (through intrusion of the upper molars). This combined use of skeletal anchorage allowed for significant dento-skeletal changes, with a favorable impact on function, esthetics, and occlusal stability (Figure 16).

### 3.3. Vertical Control Outcomes (Incisor Intrusion)

This subsection reports vertical control achieved through mini-implant–supported simultaneous intrusion of the upper and lower incisors in a deep-bite presentation (Case 4).

**Figure 17:**
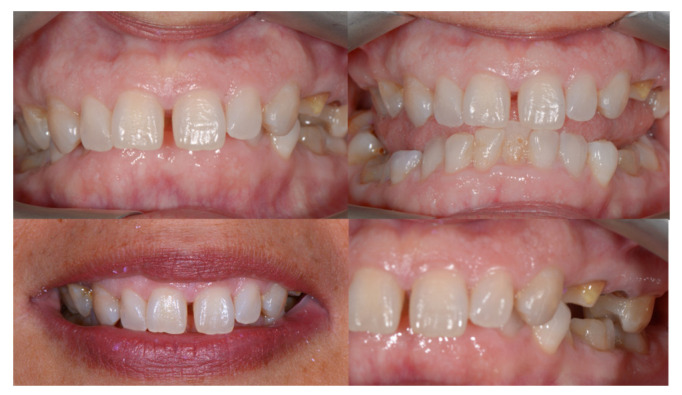
Intraoral images—initial situation.

A 48-year-old female patient (Case 4) presented to the clinic seeking a full prosthetic rehabilitation, expressing dissatisfaction with the appearance of her smile—particularly the visible diastema, pronounced gummy smile, and dental crowding. Orthodontic evaluation revealed a Class II/1 malocclusion, complete deep bite, and moderate dento-maxillary discrepancy (Figure 17).

The main therapeutic objective was the correction of the deep bite to create a stable occlusal foundation for subsequent prosthetic rehabilitation. An esthetic fixed orthodontic appliance was placed on both arches, following the standard treatment protocol. After completing the alignment and leveling phase, four orthodontic mini-implants were inserted, according to the recommendations found in the literature:

- Two vestibular mini-implants in the maxilla, positioned interradicular between teeth 12–13 and 22–23, at the junction between attached and movable mucosa, measuring 1.6 mm × 8 mm (G2, without hole);
- Two vestibular mini-implants in the mandible, positioned between teeth 32–33 and 42–43, measuring 1.4 mm × 8 mm (G2, without hole).

**Figure 18:**
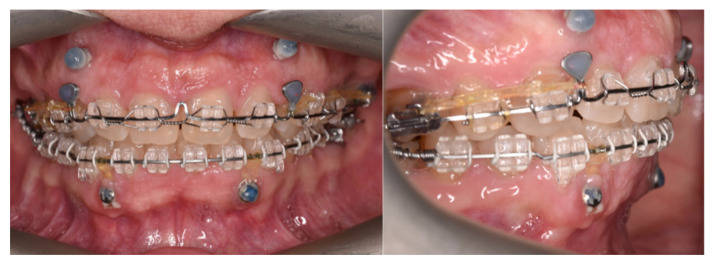
Arch appearance after intrusion, showing an optimal overbite.

It is essential that the insertion of mini-implants be performed after the leveling phase is completed to avoid interference with root movements that may occur during alignment. After insertion, the heads of the mini-implants were covered with liquid dam material to prevent irritation or injury to the vestibular mucosa. During the activation stage, elastic chains were applied from the archwire to the mini-implants, with symmetrical activation on each side of the arch to achieve equal intrusion of the anterior teeth (Figure 18). This biomechanical strategy allowed efficient and controlled correction of the vertical overbite while simultaneously reducing gingival display during smiling.

**Figure 19:**
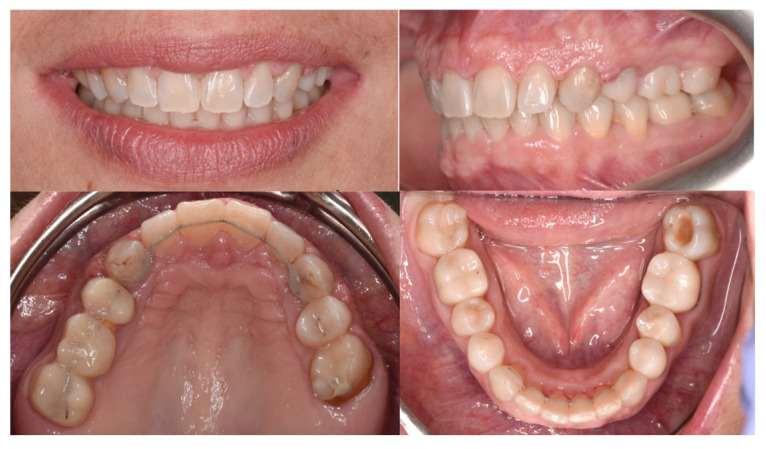
Final appearance of the arches after removal of the fixed orthodontic appliance, prosthetic rehabilitation, and placement of retainers.

At the end of the orthodontic treatment, as shown in Figure 19, the deep bite was successfully corrected, achieving an optimal overbite. In addition, the gummy smile was significantly reduced, allowing for harmonious prosthetic rehabilitation and the achievement of an esthetic and balanced smile.

## 4. Discussion

The clinical cases presented in this series illustrate the versatility and predictability of orthodontic mini-implants as skeletal anchorage devices in managing complex malocclusions across different clinical contexts. Mini-implants enable the clinician to apply controlled forces directly to the dentoalveolar or skeletal structures, minimizing dependence on dental anchorage and enhancing biomechanical efficiency. The following discussion integrates key biomechanical, clinical, and planning considerations derived from each case.

The success of orthodontic treatment and the long-term stability of mini-implants depend largely on the insertion technique, as well as on the precise selection of their placement site and dimensions. One of the main advantages of these anchorage systems is the extensive variety of sizes available, which allows for accurate adjustment to the specific anatomical and biomechanical demands of each case. This dimensional flexibility endows mini-implants with exceptional versatility, making them suitable for a wide range of orthodontic applications (i.e., intrusion, distalization, or palatal expansion), while ensuring reliable anchorage control and reducing the likelihood of mechanical or biological complications [8].

Selection of mini-implant dimensions must be tailored to each anatomical site to achieve optimal primary stability and meet specific biomechanical demands. In our series, different TAD sizes were judiciously chosen for different regions. For instance, 1.6 × 10 mm implants were inserted in the maxillary interradicular space between the second premolar and first molar to support anterior retraction, whereas a smaller 1.4 × 8 mm screw was used in the mandibular anterior region to intrude lower incisors. This site-specific approach aligns with survey data indicating that orthodontists commonly favor 1.6 × 8–10 mm screws in maxillary interradicular sites while opting for shorter, narrower implants in anatomically constrained anterior areas [9]. Studies suggest that the optimal diameter for such sites ranges between 1.5 and 1.6 mm, while the length should ensure cortical engagement without risk to adjacent roots—commonly 8–10 mm in the posterior maxilla [1,11,12].

Correct placement site coincides with known anatomical “safe zones,” as described by Poggio et al., to maximize bone support and avoid root damage [13]. Notably, our maxillary buccal insertion between the second premolar and first molar lies in a region identified as having ample interradicular bone in Poggio’s anatomical map and subsequent studies, making it ideal for a 1.6 mm diameter screw to resist retraction and intrusive forces. Similarly, the palatal mini-implants were placed where the palatal cortex is thickest (e.g., between premolar and molar regions), allowing use of larger 2.0 × 10 mm implants to withstand heavy forces for molar intrusion or molar distalization [14].

In case 3—mini-implant–assisted rapid palatal expansion (MARPE), 2 × 12 mm mini-implants were placed in a paramedian position, an area free of dental roots that provides dense cortical bone and sufficient bone volume to ensure stable and safe anchorage. The use of longer mini-implants in this region allows for bicortical engagement, which significantly enhances primary stability and resistance to dislodgment under the high transverse forces generated during expansion [15]. Precise positioning was achieved through digital planning, which enabled accurate assessment of bone thickness and angulation, minimizing the risk of perforation and ensuring symmetrical force distribution [16]. The combination of paramedian placement, bicortical engagement, and computer-guided planning thus maximizes the mechanical effectiveness and predictability of MARPE protocols, as supported by current literature on skeletal expansion techniques [17,18]. Mini-implant-assisted rapid palatal expansion (MARPE) has become a reliable non-surgical alternative to surgically assisted expansion in late adolescents and adults [19]. Compared to SARPE, MARPE offers several advantages: absence of surgical morbidity, reduced postoperative discomfort, and preservation of midpalatal integrity while still producing significant skeletal expansion verified by CBCT analysis [17]. Clinical evidence indicates that MARPE shows greater transversal skeletal changes in the midface and posterior and anterior maxillary base measurements, with superior stability and less relapse after retention [20,21].

Case 3 also exemplifies the versatility of orthodontic mini-implants, demonstrating their capacity to serve distinct biomechanical purposes—to achieve transverse skeletal expansion of the maxilla and to induce vertical changes through posterior intrusion for open-bite correction. The ability to use mini-implants to address discrepancies in both planes highlights the adaptability and efficiency of temporary anchorage devices (TADs) in comprehensive orthodontic treatment. The patient benefited from a total of six orthodontic mini-implants, which provided anchorage for both transversal and vertical correction. After the palatal expansion was obtained with the aid of two paramedian mini-implants, according to the treatment plan, the expander remained in situ as a passive retainer, with the arms for the first molars removed to avoid interference with subsequent mechanics. This decision was based on evidence showing that the midpalatal suture remains only partially remodeled even seven months after expansion, and that a retention period of approximately 12 months is necessary to allow full ossification and ensure long-term skeletal stability [22].

Since a transpalatal arch (TPA) primarily preserves dental alignment and does not effectively maintain skeletal expansion, it was not used in this case [22]. The chosen approach (i.e., maintaining the expander itself as a passive appliance for one year) allowed the preservation of the skeletal widening achieved with MARPE while simultaneously enabling posterior intrusion to address the anterior open bite. This strategy optimized treatment efficiency, avoided unnecessary appliance changes, and reduced overall treatment duration.

For the vertical component of treatment, four additional mini-implants were placed: two buccally (1.6 × 10 mm) and two palatally (2.0 × 10 mm), bilaterally between the first and second molars (6–7 region). The 6–7 interradicular area offers greater bone width and a longer moment arm compared with the 5–6 region, increasing the efficiency of intrusive forces [23,24]. While the buccal site provides easier clinical access, the palatal site presents approximately 6 mm of interradicular spacing, improving mechanical stability and reducing the risk of root interference, particularly during vertical movement [25]. Using two TADs per side (buccal and palatal) distributes the intrusive forces more evenly across the posterior segment and minimizes tipping or rotational tendencies compared with single-screw systems [26,27].

The versatility of orthodontic mini-implants lies not only in their dimensional adaptability and their ability to facilitate both tooth and skeletal changes across different planes within the same case, but also in their capacity to function as both direct and indirect anchorage throughout a single treatment sequence. In Case 1, the same vestibular mini-implants placed mesial to the maxillary first molars were initially employed for indirect anchorage to stabilize the posterior segment during canine and premolar distalization, using a segmented 0.019 × 0.025-inch stainless-steel auxiliary arch bonded directly to the teeth. Once this phase was completed, the same mini-implants were subsequently repurposed as direct anchorage units to retract the anterior segment, illustrating a smooth transition in biomechanics without additional insertion procedures. This approach offered multiple benefits, including reduced patient discomfort, simplified hygiene maintenance, and improved esthetics, since anterior bonding was deferred until the second treatment phase.

The use of direct anchorage for incisor retraction offers significant biomechanical advantages: the force vector passes closer to the center of resistance of the anterior segment, minimizing incisor tipping and preventing anchorage loss that would otherwise compromise posterior tooth position [28]. In conventional mechanics, molar anchorage loss can reduce the extraction space available for incisor retraction and lead to suboptimal overjet correction [29]. By contrast, mini-implants provide a stationary anchorage unit, enabling full retraction while preserving molar position and overall arch integrity. Furthermore, the simplified force system shortens treatment duration and improves control over torque and root angulation of the anterior teeth [30].

Similarly, in Case 2, the palatal mini-implant exhibited comparable versatility. Distalization was achieved with direct anchorage provided by a single palatal mini-implant (2 × 10 mm). The palatal site was selected to ensure the distal force passes closer to the center of resistance of the molar, thereby minimizing unwanted tipping moments and avoiding soft-tissue interference frequently associated with buccal placement. This biomechanically favorable configuration also contributes to improved vertical dimension control and more predictable molar movement [31]. Following the failure of a distalizing hook, the biomechanics were modified from direct to indirect anchorage by connecting the mini-implant to a rigid stainless-steel segment stabilizing the second premolar, thus enabling continued molar distalization through a controlled force system. This ability to seamlessly alter force direction and anchorage type using the same device underscores one of the greatest advantages of mini-implants—their biomechanical flexibility. The capacity to employ a single implant for multiple anchorage strategies increases treatment predictability, reduces procedural trauma, and exemplifies the dynamic role of temporary anchorage devices in contemporary orthodontics.

Beyond conventional labial fixed appliances, mini-implants have also been successfully combined with lingual orthodontics to support en masse distalization and camouflage treatment in selected Class II and Class III patients. Preliminary clinical evidence has shown that maxillary en masse distalization can be performed with a completely customized lingual appliance supported by miniscrew anchorage, helping clinicians reach the planned sagittal correction goals [32]. Miniscrew-assisted customized lingual appliances have also been reported to enhance anchorage control and vertical management in severe skeletal Class II cases [33]. In Class III camouflage, the use of miniscrews together with lingual brackets has been described to manage complex problems (e.g., open bite/crowding/asymmetry) when conventional mechanics would be more challenging [34]. Collectively, these reports support the versatility of mini-implants across different appliance systems, allowing clinicians to extend biomechanical possibilities and customize anchorage strategies when case selection and planning are appropriate.

Building upon this concept, Case 4 illustrates another dimension of mini-implant versatility: their use for precise vertical control in the management of deep bite through incisor intrusion. Mini-implants allow pure intrusion of the anterior segment without the side effects commonly associated with conventional mechanics, such as incisor proclination or anchorage loss, thereby extending their utility from sagittal and transverse corrections to vertical discrepancies as well [24]. Deep bite represents one of the most frequent vertical malocclusions in orthodontic practice and is often associated with excessive incisor display, gingival impingement, and occlusal trauma. Traditional correction techniques, such as the use of utility arches or reverse curve mechanics, have historically achieved bite opening by combining incisor intrusion with molar extrusion; however, these methods often lead to unwanted proclination of the incisors [35,36]. In this case, intrusion was achieved using two vestibular mini-implants in the maxilla and two in the mandible, positioned interradicularly between the lateral incisors and canines, all placed at the mucogingival junction. The mini-implants measured 1.6 × 8 mm in the maxilla and 1.4 × 8 mm in the mandible, dimensions that align with current recommendations for interradicular placement in the anterior region [9,37]. This configuration created a direct line of force acting close to the center of resistance of the anterior segment, enabling pure vertical intrusion without tipping or torque loss.

The efficacy of this approach is supported by multiple clinical studies comparing miniscrew-supported intrusion with utility-arch mechanics. Phor et al. (2018) demonstrated that both systems produced significant incisor intrusion, but root resorption was greater in the utility-arch group, highlighting the biological advantage of skeletal anchorage due to lower apical stress [38]. Similarly, Namrawy et al. (2019) found comparable amounts of overbite reduction—2.6 ± 0.8 mm (0.49 mm/month) with miniscrews and 2.9 ± 0.8 mm (0.60 mm/month) with an intrusive arch—but noted that the intrusive-arch group exhibited significantly greater incisor proclination, while the miniscrew group maintained axial control [35]. Comparable findings were reported by Aydoğdu (2012) [39] in mandibular incisor intrusion: both implant-supported and utility-arch techniques produced similar intrusion rates (0.3–0.4 mm/month at the incisal edge), but the mini-implant group achieved better molar control and reduced anchorage loss. The degree of labial tipping was also lower (≈7° vs. 8°), indicating improved control of the intrusive vector.

In the present case, simultaneous upper and lower incisor intrusion achieved a harmonious reduction of overbite and improved incisal display without compromising molar position or facial esthetics. The use of four strategically positioned mini-implants enabled direct and continuous application of vertical forces, minimized patient discomfort, and simplified biomechanics compared with conventional intrusion arches.

Overall, these outcomes align with current evidence showing that both miniscrew and utility-arch mechanics can achieve similar magnitudes of intrusion, but mini-implants provide superior control over tooth inclination, anchorage, and biological response [40,41]. Within the context of this case series, the correction of deep bite through skeletal anchorage further reinforces the versatility of mini-implants, demonstrating their capacity to offer individualized, three-dimensional control in vertical, transverse, and sagittal planes.

Although only minor soft-tissue complications related to inadequate oral hygiene were observed in the presented cases, it is important to acknowledge that miniscrew-related complications are well documented in everyday orthodontic practice. These may include insufficient primary stability, miniscrew mobility during loading, peri-implant mucosal inflammation, early implant loosening, root injury, and, in certain anatomical sites, sinus perforation [42,43,44,45,46,47]. Even when the overall failure rate of orthodontic miniscrews is relatively low, such events may significantly affect treatment progression, biomechanics, and patient comfort, and may necessitate modification of the treatment plan or reinsertion of the device [48]. Consequently, careful case selection, thorough radiographic evaluation, precise insertion technique, and appropriate loading protocols are essential to minimize risks and ensure predictable outcomes. These considerations should be an integral part of treatment planning when miniscrews are used as skeletal anchorage devices [49].

The principal strength of this study lies in the detailed clinical documentation and the comprehensive demonstration of the biomechanical versatility of orthodontic mini-implants across multiple treatment scenarios. By illustrating their application in sagittal, transverse, and vertical corrections, the present paper provides valuable insight into the adaptability and clinical decision-making processes associated with skeletal anchorage. The sequential use of the same mini-implants for both direct and indirect anchorage further exemplifies a pragmatic and minimally invasive approach, highlighting their potential for efficient, patient-centered orthodontic care. Nevertheless, several limitations must be acknowledged. The retrospective nature and limited sample size inherently restrict the generalizability of the findings and preclude statistical validation of clinical outcomes. Systematic post-treatment data were not available for all cases at the time of manuscript preparation. Therefore, the reported outcomes should be interpreted as short-term clinical observations. No definitive conclusions regarding long-term stability or relapse can be drawn, and future studies with extended follow-up are warranted. Additionally, potential operator variability and differences in anatomical conditions may influence implant stability and treatment response. Future research should therefore aim to validate these findings through prospective, controlled, and multicentric trials incorporating standardized outcome measures and long-term follow-up to assess stability and biological responses.

## 5. Conclusions

The versatility of orthodontic mini-implants represents one of their most significant clinical advantages, allowing their application across a wide spectrum of biomechanical scenarios. Their design and dimensional variability enable precise adaptation to different anatomical regions, ensuring optimal stability and safety during insertion. Mini-implants can serve as both direct and indirect anchorage units, be incorporated into various mechanics such as retraction, intrusion, distalization, or expansion, and can be repositioned or repurposed as treatment progresses. This adaptability not only enhances the efficiency and predictability of orthodontic therapy but also reduces the need for additional auxiliary devices, reinforcing the role of mini-implants as a cornerstone of modern, minimally invasive anchorage strategies. The clinician can dynamically adjust the mechanics—from direct to indirect anchorage or from unilateral to bilateral force systems—according to treatment progression. This adaptability is one of the defining strengths of mini-implants, supporting individualized, minimally invasive treatment strategies. In conclusion, mini-implants offer a safe, effective, and efficient means of achieving the desired tooth movements in high-anchorage cases, outperforming conventional anchorage systems in both magnitude of tooth movement and control of side effects.

## Figures and Tables

**Table 1:** Baseline characteristics and miniscrew-related information of the presented cases.

Case	Age (Years)	Sex	Skeletal/Dental Diagnosis	Main Treatment Objective	Miniscrew Location	Miniscrew Dimensions (mm)	Intended Tooth Movement	Treatment Status
1	34	M	Skeletal Class II hypodivergent pattern, Dental Class II, division II	Orthodontic camouflage of Class II malocclusion using skeletal anchorage	Vestibular Maxilla	1.6 × 10	Retraction of upper incisors	Completed
2	27	M	Skeletal Class I, hyperdivergent pattern, Dental Class II	Correction of Open Bite and expansion of narrow maxilla	Paramedian Palatal Vestibular	2 × 12 2 × 10 1.6 × 10	Palatal expansion Molar intrusion	Ongoing
3	48	F	Skeletal Class II, hypodivergent pattern, Dental Class II	Correction of Deep Bite, Orthodontic camouflage of Class II malocclusion	Anterior Vestibular Maxilla Anterior Vestibular Mandible	1.6 × 8 1.4 × 8	Upper and lower incisor intrusion	Completed
4	22	F	Skeletal class II, normodivergent pattern, Dental Class I	Opening space for impacted canine	Palatal	2 × 10	Upper molar unilateral distalisation	Ongoing

M, male; F, female.

**Table 2:** Miniscrew-related clinical parameters.

Case	Insertion Site	Mini-Implant Dimensions (mm)	Insertion Torque/Speed	Duration of Loading
1	Vestibular maxilla	1.6 × 10	10 Ncm/20 rpm	9 months
2	Paramedian	2 × 12	25 Ncm/30 rpm	3 months
	Palatal	2 × 10	20 Ncm/30 rpm	5 months
	Vestibular Maxilla	1.6 × 10	10 Ncm/20 rpm	5 months
3	Anterior Vestibular Maxilla	1.6 × 8	10 Ncm/20 rpm	6 months
	Anterior Vestibular Mandible	1.4 × 8	10 Ncm/15 rpm	6 months
4	Palatal	2 × 10 mm	20 Ncm/30 rpm	8 months

## Data Availability

The original contributions presented in this study are included in the article. Further inquiries can be directed to the corresponding authors.

## Abbreviations

The following abbreviations are used in this manuscript:

GDPR	General Data Protection Regulation
CBCT	Cone Beam Computed Tomography
TAD	Temporary Anchorage Device
MARPE	Mini-implant Assisted Rapid Palatal Expansion
TPA	Transpalatal Arch
SARPE	Surgically Assisted Rapid Palatal Expansion

## References

[1] Jong S.L., Kook K.J., Park Y.-C., Vanarsdall R.L. (2008). Applications of Orthodontic Mini-Implants.

[2] Dhayal J., Ria S., Govind H.G., Sangita K., Vaishnavi J., Sharda B. (2023). Mini Implants in Orthodontics—A Review. J. Adv. Med. Dent. Sci. Res..

[3] Proffit W.R., Fields H., Larson B., Sarver D.M. (2018). Contemporary Orthodontics.

[4] Bucur S.-M., Vaida L.L., Olteanu C.D., Checchi V. (2021). A Brief Review on Micro-Implants and Their Use in Orthodontics and Dentofacial Orthopaedics. Appl. Sci..

[5] Currier F., Kadioglu O. (2013). The Orthodontist’s Role in MDI Therapeutics: ORTHO Transitional Anchorage Devices (TADs) and Related Applications. Mini Dental Implants: Principles and Practice.

[6] Umalkar S.S., Jadhav V.V., Paul P., Reche A. (2022). Modern Anchorage Systems in Orthodontics. Cureus.

[7] Panaite T., Balcos C., Savin C., Olteanu N.D., Karvelas N., Romanec C., Vieriu R.M., Chehab A., Zetu I. (2024). Exploring the Use, Perceptions, and Challenges of Mini-Implants in Orthodontic Practice: A Survey Study. Front. Oral Health.

[8] Jing Z., Wu Y., Jiang W., Zhao L., Jing D., Zhang N., Cao X., Xu Z., Zhao Z. (2016). Factors Affecting the Clinical Success Rate of Miniscrew Implants for Orthodontic Treatment. Int. J. Oral Maxillofac. Implant..

[9] Bungau T.C., Vaida L., Moca A., Buhas C.L., Talpos-Niculescu C.I., Pacurar M., Craciun D., Popa M. (2025). Preferences in mini-implant dimensions among romanian orthodontists. Rom. J. Oral Rehabil..

[10] WMA Declaration of Helsinki—Ethical Principles for Medical Research Involving Human Subjects. https://www.wma.net/policies-post/wma-declaration-of-helsinki-ethical-principles-for-medical-research-involving-human-subjects/.

[11] Motoyoshi M. (2011). Clinical Indices for Orthodontic Mini-Implants. J. Oral Sci..

[12] Luzi C., Verna C., Melsen B. (2009). Guidelines for Success in Placement of Orthodontic Mini-Implants. J. Clin. Orthod..

[13] Poggio P.M., Incorvati C., Velo S., Carano A. (2006). “Safe Zones”: A Guide for Miniscrew Positioning in the Maxillary and Mandibular Arch. Angle Orthod..

[14] Chaimanee P., Suzuki B., Suzuki E.Y. (2011). “Safe Zones” for Miniscrew Implant Placement in Different Dentoskeletal Patterns. Angle Orthod..

[15] Liu P.-H., Chen Y.-F., Pan C.-Y., Sheen M.-H., Chen B.-S., Chang H.-P. (2022). Maxillary Skeletal Expansion with Monocortical and Bicortical Miniscrew Anchorage: A 3D Finite Element Study. Appl. Sci..

[16] Lee R.J., Moon W., Hong C. (2017). Effects of Monocortical and Bicortical Mini-Implant Anchorage on Bone-Borne Palatal Expansion Using Finite Element Analysis. Am. J. Orthod. Dentofac. Orthop..

[17] Suzuki H., Moon W., Previdente L.H., Suzuki S.S., Garcez A.S., Consolaro A. (2016). Miniscrew-Assisted Rapid Palatal Expander (MARPE): The Quest for Pure Orthopedic Movement. Dent. Press J. Orthod..

[18] Villa-Obando Y.A., Correa-Osorio S.M., Castrillon-Marin R.A., Vivares-Builes A.M., Ardila C.M. (2024). Effect of Anchorage Modifications on the Efficacy of Miniscrew-Assisted Rapid Palatal Expansion: A Systematic Review and Meta-Analysis. Cureus.

[19] Hoque T., Srinivasan D., Gnaneswar S., Chakravarthi S., Rajaram K. (2021). Microimplant Assisted Rapid Palatal Expansion: A Comprehensive Review. J. Clin. Diagn. Res..

[20] de Oliveira C.B., Ayub P., Ledra I.M., Murata W.H., Suzuki S.S., Ravelli D.B., Santos-Pinto A. (2021). Microimplant Assisted Rapid Palatal Expansion vs Surgically Assisted Rapid Palatal Expansion for Maxillary Transverse Discrepancy Treatment. Am. J. Orthod. Dentofac. Orthop..

[21] Cantarella D., Dominguez-Mompell R., Moschik C., Mallya S.M., Pan H.C., Alkahtani M.R., Elkenawy I., Moon W. (2018). Midfacial Changes in the Coronal Plane Induced by Microimplant-Supported Skeletal Expander, Studied with Cone-Beam Computed Tomography Images. Am. J. Orthod. Dentofac. Orthop..

[22] Prado G.P.R., Furtado F., Aloise A.C., Biló J.P.R., Masako Ferreira L., Pereira M.D. (2014). Stability of Surgically Assisted Rapid Palatal Expansion with and without Retention Analyzed by 3-Dimensional Imaging. Am. J. Orthod. Dentofac. Orthop..

[23] Kim H.-J., Yun H.-S., Park H.-D., Kim D.-H., Park Y.-C. (2006). Soft-Tissue and Cortical-Bone Thickness at Orthodontic Implant Sites. Am. J. Orthod. Dentofac. Orthop..

[24] Paik C.-H., Park O.-K., Woo Y., Kim T.-W. (2012). Miniscrew Implant Anchorage for Intrusion of Teeth. Orthodontic Miniscrew Implants: Clinical Applications.

[25] Paik C.-H., Park O.-K., Woo Y., Kim T.-W. (2012). Anatomic Considerations and Placement/Removal of Orthodontic Miniscrew Implants. Orthodontic Miniscrew Implants: Clinical Applications.

[26] Patel S.D., Ghosh A., Parashar P., Shenavi L., Agarwal S.K., Rawat S., Makkad R.S. (2024). Effectiveness of Miniscrew-Supported Molar Intrusion: A Clinical Study. J. Pharm. Bioallied Sci..

[27] Kravitz N.D., Kusnoto B., Tsay T.P., Hohlt W.F. (2007). The Use of Temporary Anchorage Devices for Molar Intrusion. J. Am. Dent. Assoc..

[28] Papadopoulos M.A., Tarawneh F. (2013). Miniscrew Implants for Temporary Skeletal Anchorage in Orthodontic Treatment. Skeletal Anchorage in Orthodontic Treatment of Class II Malocclusion. Contemporary Applications of Orthodontic Implants, Miniscrew Implants and Mini Plates.

[29] Becker K., Pliska A., Busch C., Wilmes B., Wolf M., Drescher D. (2018). Efficacy of Orthodontic Mini Implants for En Masse Retraction in the Maxilla: A Systematic Review and Meta-Analysis. Int. J. Implant Dent..

[30] Kuc A.E., Kotuła J., Nahajowski M., Warnecki M., Lis J., Amm E., Kawala B., Sarul M. (2022). Methods of Anterior Torque Control during Retraction: A Systematic Review. Diagnostics.

[31] Papadopoulos M.A. (2020). Efficient Distalization of Maxillary Molars with Temporary Anchorage Devices for the Treatment of Class II Malocclusion. Turkish J. Orthod..

[32] Beyling F., Klang E., Niehoff E., Schwestka-Polly R., Helms H.-J., Wiechmann D. (2021). Class II Correction by Maxillary En Masse Distalization Using a Completely Customized Lingual Appliance and a Novel Mini-Screw Anchorage Concept—Preliminary Results. Head Face Med..

[33] Wang X.-D., Lei F., Liu D.-W., Zhang J.-N., Liu W., Song Y., Zhou Y.-H. (2017). Miniscrew-Assisted Customized Lingual Appliances for Predictable Treatment of Skeletal Class II Malocclusion with Severe Deep Overbite and Overjet. Am. J. Orthod. Dentofac. Orthop..

[34] Yanagita T., Kuroda S., Takano-Yamamoto T., Yamashiro T. (2011). Class III Malocclusion with Complex Problems of Lateral Open Bite and Severe Crowding Successfully Treated with Miniscrew Anchorage and Lingual Orthodontic Brackets. Am. J. Orthod. Dentofac. Orthop..

[35] El Namrawy M.M., El Sharaby F., Bushnak M. (2019). Intrusive Arch versus Miniscrew-Supported Intrusion for Deep Bite Correction. Open Access Maced. J. Med. Sci..

[36] Millett D.T., Cunningham S.J., O’Brien K.D., Benson P.E., de Oliveira C.M. (2018). Orthodontic Treatment for Deep Bite and Retroclined Upper Front Teeth in Children. Cochrane Database Syst. Rev..

[37] Romanec C.L., Panaite T., Zetu I.N. (2025). Dimensions Define Stability: Insertion Torque of Orthodontic Mini-Implants: A Comparative In Vitro Study. J. Clin. Med..

[38] Phor D., Sharma A., Upadhyay S., Sharma A., Vaidya A. (2018). Comparison of Intrusive Effects of Miniscrews and Utility Arch and Their Effects on Root Resorption. IOSR J. Dent. Med. Sci..

[39] Aydoğdu E., Özsoy Ö.P. (2011). Effects of Mandibular Incisor Intrusion Obtained Using a Conventional Utility Arch vs Bone Anchorage. Angle Orthod..

[40] Al-Sibaie S., Hajeer M.Y. (2014). Assessment of Changes Following En-Masse Retraction with Mini-Implants Anchorage Compared to Two-Step Retraction with Conventional Anchorage in Patients with Class II Division 1 Malocclusion: A Randomized Controlled Trial. Eur. J. Orthod..

[41] Upadhyay M., Yadav S., Patil S. (2008). Mini-Implant Anchorage for En-Masse Retraction of Maxillary Anterior Teeth: A Clinical Cephalometric Study. Am. J. Orthod. Dentofac. Orthop..

[42] Kuroda S., Tanaka E. (2014). Risks and Complications of Miniscrew Anchorage in Clinical Orthodontics. Jpn. Dent. Sci. Rev..

[43] Kravitz N.D., Kusnoto B. (2007). Risks and Complications of Orthodontic Miniscrews. Am. J. Orthod. Dentofac. Orthop..

[44] Papageorgiou S.N., Zogakis I.P., Papadopoulos M.A. (2012). Failure Rates and Associated Risk Factors of Orthodontic Miniscrew Implants: A Meta-Analysis. Am. J. Orthod. Dentofac. Orthop..

[45] Ichinohe M., Motoyoshi M., Inaba M., Uchida Y., Kaneko M., Matsuike R., Shimizu N. (2019). Risk Factors for Failure of Orthodontic Mini-Screws Placed in the Median Palate. J. Oral Sci..

[46] Cheng S.-J., Tseng I.-Y., Lee J.-J., Kok S.-H. (2004). A Prospective Study of the Risk Factors Associated with Failure of Mini-Implants Used for Orthodontic Anchorage. Int. J. Oral Maxillofac. Implant..

[47] Casaña-Ruiz M.D., Bellot-Arcís C., Paredes-Gallardo V., García-Sanz V., Almerich-Silla J.M., Montiel-Company J.M. (2020). Risk Factors for Orthodontic Mini-Implants in Skeletal Anchorage Biological Stability: A Systematic Literature Review and Meta-Analysis. Sci. Rep..

[48] Tarigan S.H.P., Sufarnap E., Bahirrah S. (2024). The Orthodontic Mini-Implants Failures Based on Patient Outcomes: Systematic Review. Eur. J. Dent..

[49] Bungău T.C., Moca A.E., Ciavoi G., Romanul I.M., Vaida L.L., Buhaș C.L. (2024). Usage and Preferences of Orthodontic Mini-Implants Among Romanian Practitioners: A Survey Study. Dent. J..

